# Mass Spectrometry and ^1^H-NMR Study of *Schinopsis lorentzii* (Quebracho) Tannins as a Source of Hypoglycemic and Antioxidant Principles

**DOI:** 10.3390/molecules25143257

**Published:** 2020-07-17

**Authors:** Nunzio Cardullo, Vera Muccilli, Vincenzo Cunsolo, Corrado Tringali

**Affiliations:** Department of Chemical Sciences, University of Catania, Viale A. Doria 6, 95125 Catania, Italy; ncardullo@unict.it (N.C.); vcunsolo@unict.it (V.C.); ctringali@unict.it (C.T.)

**Keywords:** tannins, α-glucosidase, α-amylase, DPPH, ORAC, mass spectrometry, ^1^H-NMR

## Abstract

The ethyl acetate extract of the commercial tannin Tan’Activ QS-SOL (from *Schinopsis lorentzii* wood), employed for the production of red wine, was subjected to chromatography on Sephadex LH-20, providing nine fractions (A-1–A-9), which were estimated for total phenols content (GAE), antioxidant activity (DPPH, ORAC), and hypoglycemic activity (α-glucosidase and α-amylase inhibition). All the fractions were analyzed by means of HPLC/ESI-MS/MS and ^1^H-NMR to identify the principal active constituents. Fractions A-1 and A-3 showed the highest antioxidant activity and gallic acid (**1**), pyrogallol (**3**), eriodictyol (**6**), catechin (**12**), and taxifolin (**30**) were identified as the major constituents. The highest α-glucosidase and α-amylase inhibitory activity was observed in fractions A-7–A-9 containing condensed (**9′**, **15**, **18**, **19**, **23**, and **27**) hydrolysable tannins (**13** and **32**) as well as esters of quinic acid with different units of gallic acid (**5**, **11**, **11′**, **14**, and **22**). This last class of gallic acid esters are here reported for the first time as α-glucosidase and α-amylase inhibitors.

## 1. Introduction

Diabetes mellitus (DM) is a chronic metabolic disorder with an increasing global prevalence and incidence at an alarming rate. It is characterized by insulin hormone dysfunction and a resulting high blood glucose level (hyperglycemia). There are several different types of diabetes, the most common form in the general population being type 2 diabetes (non-insulin-dependent), mostly affecting adults and accounting for about 90% of all cases of diabetes. Obesity, aging, and familial history of diabetes have been identified as significant risk factors. Metabolic complications such as cardiovascular disease, angiopathy, neuropathy, nephropathy and others are frequently associated with Type 2 diabetes or can cause it to worsen [1]. Moreover, hyperglycemia increases reactive oxygen species’ production, causing oxidative tissue damage [2].

Several oral antidiabetic drugs such as biguanides, meglitinide, sulfonylureas, thiazolidinedione, as well as inhibitors of dipeptidyl peptidase 4, sodium-glucose cotransporter, and carbohydrate hydrolyzing enzymes have been employed to manage postprandial hyperglycemia [3]. Among all the commercial antidiabetic drugs, α-glucosidase (α-GLU) and α-amylase (α-AMY) inhibitors are the most effective in reducing postprandial hyperglycemia [4]. The commercial inhibitors—acarbose, voglibose, and miglitol—are pseudo-carbohydrates that competitively inhibit these metabolic enzymes, also causing delayed digestion; thus, the absorption of carbohydrates is reduced and this prevents postprandial hyperglycemia [4]. These inhibitors taken before meals rich in complex carbohydrates, reduce the glycated hemoglobin (HbA1c) levels but have a relatively high rate of discontinuation, owing to the occurrence of numerous gastrointestinal side effects [5].

For these reasons, several researchers are dedicated to the discovery of new α-glucosidase and/or α-amylase inhibitors with fewer or absent undesired effects. Nowadays, there is a renewed interest in plant-based medicines and functional foods for the prevention and cure of diabetes and obesity, given their slight or absent side effects [6]. The plant kingdom is a promising source of bioactive products with hypoglycemic activity [7,8]. Among natural products, some polyphenols occurring in edible plants showed interesting α-glucosidase and/or α-amylase inhibitory activity [8]. Moreover, the well-known antioxidant properties of polyphenols represent a further advantage in the search for potential dual-action antidiabetic agents, able to join hypoglycemic properties and reduction in oxidative damage associated with diabetes complications [2]. Consequently, several analogues of natural polyphenols have been evaluated as hypoglycemic agents, namely stilbenoid glycosides [9], bisphenol neolignans [10], rosmarinic acid amides [11], 3,4-dihydroxypyrrolidine-based compounds [12], and biscoumarins [13]. 

Among plant polyphenols, tannins raise considerable interest for several biological properties, including antidiabetic activity [14]. According to their chemical structures, tannins can be divided into (1) condensed tannins (proanthocyanidins, PACs), (2) hydrolyzable tannins (mainly ellagitannins and gallotannins), and (3) phlorotannins [15]. We have recently evaluated a selection of hydrolyzable tannins, such as *C*-glucosidic ellagitannins and galloylated glucoses, as α-GLU and α-AMY inhibitors [16]. Moreover, we have studied oenological commercial hydrolyzable tannins provided by Silvateam Spa (http://en.silvateam.com) and thanks to an extraction/fractionation procedure suitable for industrial applications, we succeeded in obtaining polyphenol-enriched fractions with higher antioxidant and hypoglycemic activity than those of the corresponding extracts [17,18]. 

In light of the obtained promising results, we extended the search of potential α-glucosidase and/or α-amylase inhibitors to the investigation of a commercial tannins extract from *Schinopsis lorentzii* (Griseb.) Engler (*Schinopsis quebracho-colorado Schltdl.*), a tree growing in Argentina, Paraguay, Brazil, and Bolivia. Dried quebracho extract contains about 95% proanthocyanidins, and the remaining 5% is mainly constituted of water-soluble sugars. Quebracho tannin extract is authorized by the European Union (EU Community Register of Feeds Additives) as an additive for feedstuffs [19]. Moreover, quebracho tannin extracts are used to produce all types of leather and especially, natural vegetable-processed leather. 

Thus, in this work we present the results of an assay-guided fractionation of Tan’Activ QS-SOL, a commercial tannin extract from *Schinopsis lorentzii* wood, aimed to obtain polyphenol-enriched fractions with antioxidant and/or hypoglycemic activity, the latter obtained through inhibition of α-GLU and/or α-AMY. The main constituents of the fractions were identified by the combined use of HPLC/ESI-MS/MS and ^1^H-NMR.

## 2. Results and Discussion

### 2.1. Extraction and Fractionation of Tan’Activ QS-SOL

**Figure 1:**
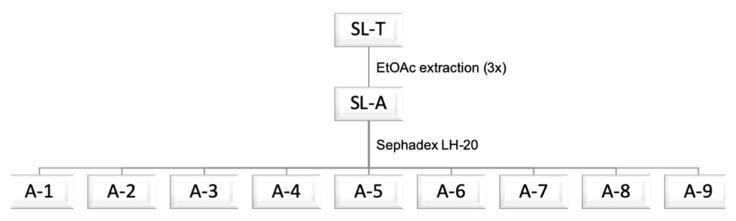
Extraction and fractionation flow chart.

As a continuation of our previous studies on tannins as potential functional food ingredients with antidiabetic and antioxidant properties [17,18], we report here a study on *Schinopsis lorentzii* (Quebracho) tannins as a source of hypoglycemic and antioxidant principles; in this work, we assessed the hypoglycemic activity of the extract and fractions by also evaluating the α-amylase inhibitory activity. A sample of Tan’Activ QS-SOL (*Schinopsis lorentzii* wood, SL-T) was extracted with ethyl acetate (EtOAc). The crude extract (SL-A) was subjected to chromatographic separation on a Sephadex-LH20 column and the eluate was pooled in nine subfractions, A-1–A-9, following a preliminary analysis performed via TLC (Figure 1, See Section 3.5 for details). A first elution was carried out with water to remove the possible presence of salts and low molecular weight sugars, or other more hydrophilic compounds contained in the SL-A extract. Subsequently, the elution was carried out with a gradient of MeOH in water and finally, with acetone in the attempt to separate the hydrolyzable tannins from condensed tannins, exploiting their different affinity with the stationary phase [20,21].

In Table 1, we report the percentage yield of SL-A (referred to SL-T powder) and of each fraction (with respect to the total eluate recovered from the Sephadex LH-20 column).

### 2.2. Antioxidant Activity and α-Glucosidase and α-Amylase Inhibition for SL-T, SL-A and A-1–A-9 Fractions

Aliquots of the commercial tannin sample (SL-T), the crude extract SL-A, and the fractions A-1–A-9 were evaluated for their antioxidant and hypoglycemic activity. Table 1 reports a) the total phenols content (measured as gallic acid equivalents (GAE), as mg/g); b) the antioxidant activities measured as scavenging of the DPPH• radical (expressed as SC_50_ in μg/mL) and Oxygen Radical Absorbance Capacity (ORAC, expressed as μmol TE/g); c) the hypoglycemic activity evaluated as α-GLU and α-AMY inhibition (expressed as IC_50_ in μg/mL). In this study, quercetin (Que) was employed as a positive control for antioxidant activity and α-GLU and α-AMY inhibition. The antidiabetic drug acarbose (Aca) was employed as the standard for α-GLU and α-AMY inhibition. 

The SL-A extract shows an antioxidant activity (DPPH SC_50_ value of 5.5 μg/mL; ORAC = 1410.7 µmol TE/g) and phenols content (GAE = 316.3 mg/g) higher than those of the commercial sample SL-T (DPPH SC_50_ = 7.4 μg/mL, ORAC = 1075.4 µmol TE/g), indicating that EtOAc selectively extracts the antioxidant polyphenols present in SL-T. Moreover, SL-A proved to be an effective inhibitor of both α-GLU and α-AMY (IC_50_ = 6.3 μg/mL and 86.1 μg/mL, respectively), showing higher activity than the commercial sample SL-T (IC_50_ = 48.9 μg/mL and 129.3 μg/mL, respectively). Furthermore, the α-GLU inhibitory activity of SL-A is comparable to that of QUE (IC_50_ = 5.5 μg/mL) and by far superior to that of the antidiabetic drug acarbose (IC_50_ = 97.2 μg/mL). Thus, the EtOAc extraction allowed us to concentrate, in SL-A, the constituents of SL-T responsible for both antioxidant and hypoglycemic activity.

The fractions A-1–A-9 have DPPH SC_50_ values between 4.0 and 8.1 µg/mL, not far from that of QUE. Only two fractions, namely A-1 (DPPH SC_50_ = 4.0 µg/mL, ORAC = 3345.4 µmol TE/g) and A-3 (DPPH SC_50_ = 4.9 µg/mL, ORAC = 1895.1 µmol TE/g), show antioxidant activity higher than that of SL-A. Consistently with these data, the polyphenol content of fractions A-1 and A-3 is significantly higher than that of SL-A (A-1 GAE = 867.5 mg/g; A-3 GAE = 756.4 mg/g) resulting in the enrichment in phenols, respectively, of 174% (A-1) and 139% (A-3), with respect to the crude extract. It is also worth mentioning that these two fractions account for only 10.3% of the crude extract and may result useful as antioxidant additives for agro-food applications. Nevertheless, both fractions showed a poor inhibition against both α-GLU and α-AMY, and consequently, these are not the best candidates as potential dual-action antidiabetic agents. 

Fraction A-2 shows antioxidant activity and α-GLU inhibition lower than those corresponding to the extract SL-A. Conversely, this fraction resulted in a moderate α-AMY inhibitory activity. Fractions A-4 and A-5, accounting for about 26% of the total eluate, exhibited lower antioxidant (DPPH and ORAC) and inhibitory activities (towards α-GLU and α-AMY) than those observed for SL-A. Fraction A-6 (15% of the total eluate) showed antioxidant activity comparable to that of SL-A and exerted some inhibition of α-GLU (IC_50_ = 8.9 µg/mL) and α-AMY (IC_50_ = 72.5 µg/mL), although the inhibitory activities were lower than those of SL-A. 

The most promising results were observed for fractions A-7–A-9. These fractions showed fairly good antioxidant activity, a potent inhibitory activity towards α-GLU (IC_50_ in the range 2.1–3.6 µg/mL), and a moderate α-AMY inhibitory activity (IC_50_ in the range 64.2–93.6 µg/mL). In particular, fraction A-8, constituting only 5.6% of the total eluate of the column and 1.9% of the commercial tannin SL-T, exhibited the most potent inhibitory activity against α-GLU (IC_50_ = 2.1 µg/mL), much greater than that of the crude tannin SL-T (IC_50_ = 48.9 µg/mL), three times more active than that of SL-A (IC_50_ = 6.3 µg/mL), as well as 50 times higher than that of acarbose (IC_50_ = 64.2 µg/mL). The moderate inhibition of α-AMY observed for fractions A-8–A-9 may result in an advantage, because in the development of antidiabetic drugs, a higher incidence of undesirable effects has been associated with strong α-amylase inhibition [22]. Consequently, for therapeutic purposes, a potent α-GLU inhibitor with moderate inhibitory activity against α-AMY is usually preferred. In light of these results, fractions A-7 and A-8 might be employed as dual-action antidiabetic agents without further purification of their constituents.

### 2.3. Correlation Analysis

Pearson’s correlation analysis was carried out to evaluate direct correlation analysis among GAE and antioxidant activity (DPPH and ORAC) of SL-T, SL-A, and A-1–A-9 fractions. For DPPH, lower IC_50_ values mean higher activities. In this case, all IC_50_ values were converted into 1/IC_50_ values. 

DPPH and ORAC scavenging activity were highly correlated with GAE (GAE vs. DPPH: *R* = 0.0886; GAE vs. ORAC: *R* = 0.857; DPPH vs. ORAC: *R* = 0.881; *p* < 0.001). 

### 2.4. Principal Component Analysis (PCA)

**Figure 2:**
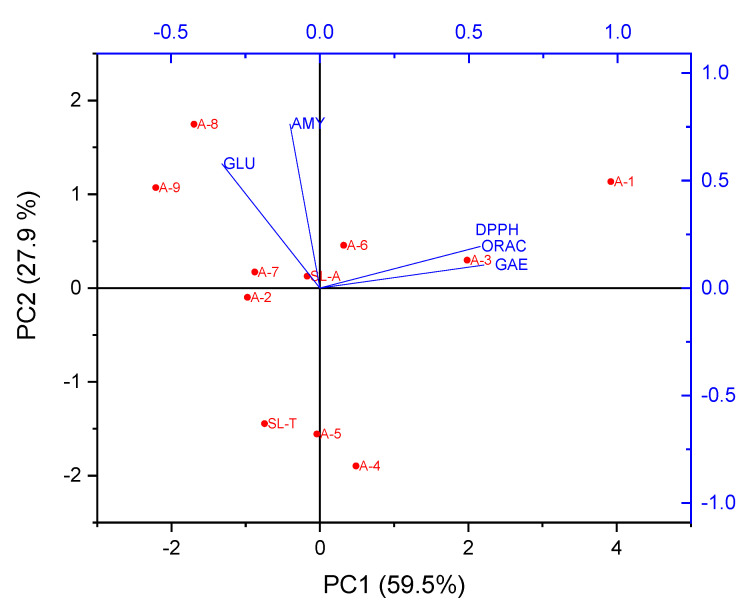
Biplot representation on the factor-plane (PC1 vs. PC2), showing vector distribution of GAE, DPPH, ORAC, α-GLU, and α-AMY within score plot of the SL-T, SL-A and fraction A-1–A-9.

Principal Component Analysis was conducted to get a general overview of the data distribution; thus, principal components (PCs) were generated. PCA based on the corresponding dataset of SL-T, SL-A, and fractions A-1–A-9, including GAE, antioxidant (DPPH and ORAC), and α-GLU and α-AMY inhibitory values, was carried out (Figure 2).

The first principal component (PC1) has the highest eigenvalue of 2.97 and accounted for 59.46% of the variability in the dataset. The second, third, and fourth PCs (PC2, PC3, and PC4) had eigenvalues of 1.39, 0.40, and 0.14 and explained 27.90%, 8.00%, and 2.79% of the variance in the data, respectively. Subsequently, by plotting the scores of the samples in the subspaces PC1 vs. PC2 (87.36% of the total variance of the data), a clear grouping of samples was observable. PCA confirms the previous observations, allowing the discrimination of different fractions around the PC1 and PC2 axes’ components and activities (Figure 2). These axes’ components correlate fractions A-1 and A-3 with antioxidant activity (DPPH and ORAC) and total phenolic content (GAE); fractions A-7–A-9 were correlated with α-GLU and α-AMY inhibitory activities. Extracted eigenvectors are reported in Table 2. The bigger the eigenvectors, the higher the correlations between variables and PCs. DPPH, ORAC, and GAE were positively associated with PC1, while α-GLU and α-AMY were positively associated with PC2.

### 2.5. Mass Spectrometric and ^1^H-NMR Analysis of A-1–A-9 Fractions

**Figure 3:**
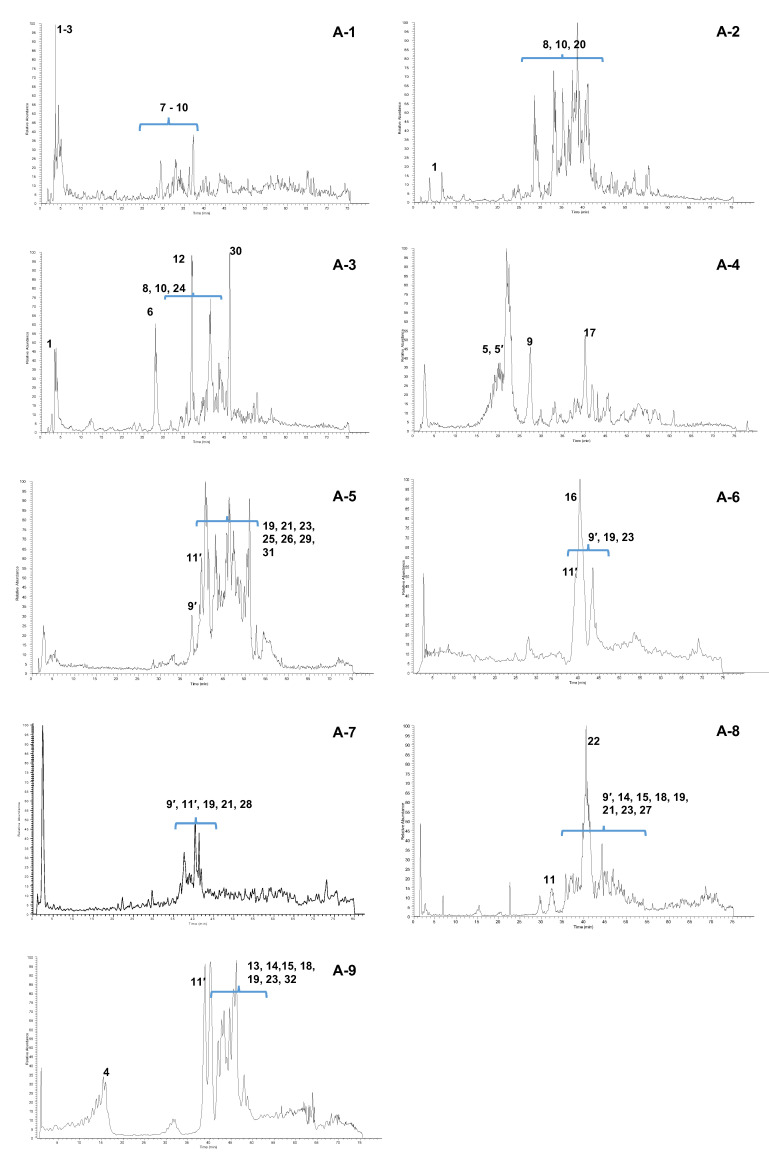
HPLC/ESI-MS/MS (TIC profiles) of A-1–A-9 fractions obtained from a *Schinopsis lorentzii* tannin.

**Figure 4:**
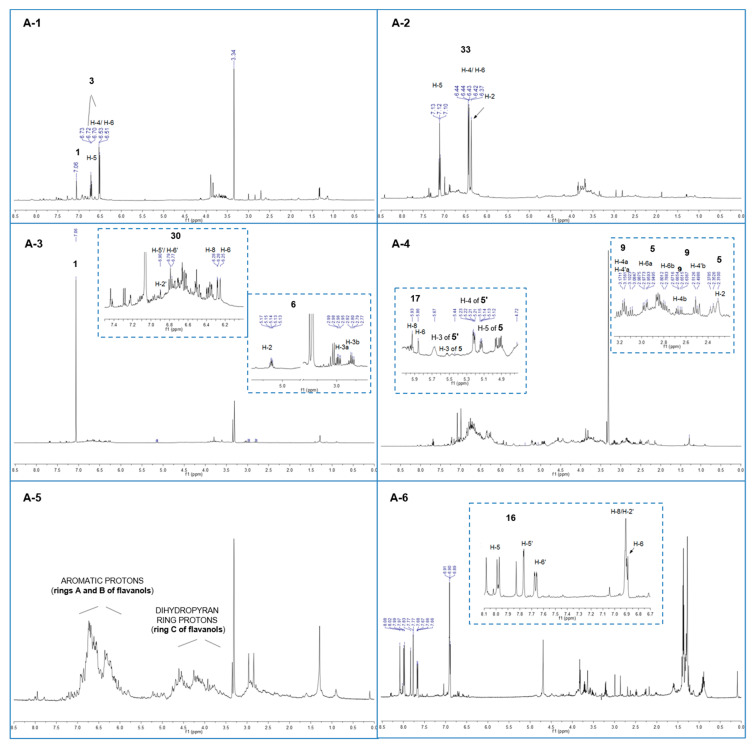
^1^H-NMR spectra (500 MHz, CD_3_OD or D_2_O) of fractions A-1–A-6.

**Figure 5:**
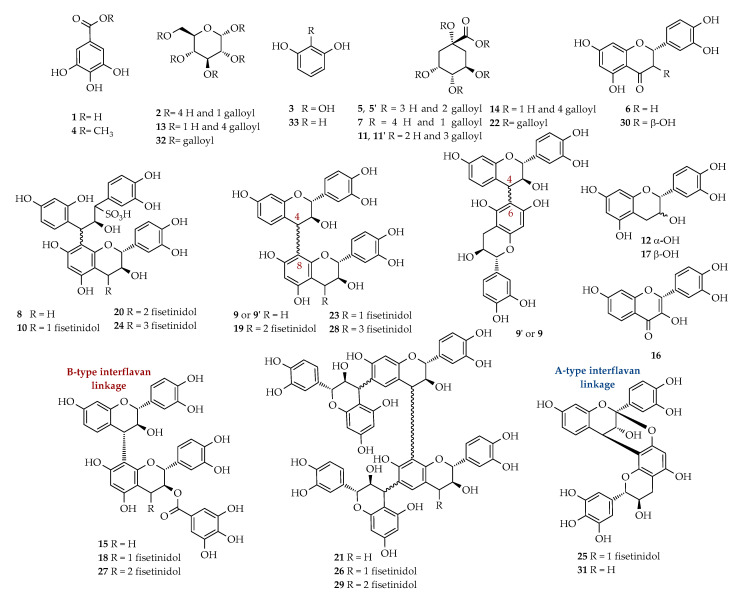
Chemical structures of identified compounds.

HPLC/ESI-MS/MS analysis was performed on the fractions A-1–A-9 to obtain useful data for the identification of the main constituents of each fraction. Figure 3 reports the total ion current (TIC) chromatograms of these fractions. A first tentative identification of the main constituents was based on the comparison of the parent ions and fragmentation spectra with literature data [23,24]; when possible, these assumptions were corroborated by ^1^H-NMR spectra (Figure 4). The constituents are numbered according to their chromatographic elution times, as listed in Table 3. For each compound, the [M − H]^−^ *m*/*z* value, the main MS/MS fragments, and where available, the ^1^H-NMR assignments were reported. The structures of all the identified compounds are reported in Figure 5.

The TIC chromatogram of fraction A-1 shows the presence of an intense and polar peak eluting between 3 and 5 min and a group of low intensity peaks between 27 and 40 min. At t_R_ = 3.1 min, a compound was detected with [M − H]^−^ at *m*/*z* 169, whose tandem mass spectrum showed a fragment at *m*/*z* 125 originating from the loss of CO_2_ (M-44). This was identified with gallic acid (**1**) and its identification was corroborated by the analysis of ^1^H-NMR spectrum of the A-1 fraction (Figure 4), showing an intense singlet at 7.06 ppm easily attributable to the aromatic protons of **1**. Gallic acid, a typical constituent of gallotannins, is reported as an effective antioxidant but a weak inhibitor of α-glucosidase and this finding is in agreement with the results reported in Table 3 [25]. The identification of gallic acid was supported by the HPLC-UV profile of the fraction A-1 compared to a standard solution (Figure S1 of Supplementary Material). The peak at T_R_ = 3.2 min gave a [M − H]^−^ at *m*/*z* 331 and it was identified as monogalloyl glucose isomer (**2**), according to its MS/MS spectrum, showing signals at *m*/*z* 271 (M-H-60) and *m*/*z* 241 (M-H-90), both arising from the fragmentation of the glucosidic ring [26] and the fragment at *m*/*z* 169 (M-H-162) ascribable to the loss of a glucose unit (Figure S2 of Supplementary Material). 

The positional isomers of compound **2** cannot be discriminated by MS data; therefore, this compound and the other molecules with similar indistinguishable structures are indicated in Table 3 as ‘isomer’. The peak at t_R_ = 3.5 min presented a [M − H]^−^ at *m*/*z* 125 and was attributed to pyrogallol (**3**), most likely deriving from the thermal decomposition of hydrolysable tannins [27]. This identification was corroborated by the ^1^H-NMR spectrum, showing two coupled signals—a triplet at 6.72 ppm (*J* = 8.1 Hz, H-5) and a doublet at 6.52 ppm (*J* = 8.1 Hz, H-4/H-6)—attributable to the A_2_X spin system of a 1,2,3-trisubstituted aromatic ring as for **3**. The peak at t_R_ = 28.0 min was due to the elution of a compound with [M − H]^−^ at *m*/*z* 343 corresponding to an isomer of monogalloyl quinic acid (**7**), whose fragmentation pattern (Figure S3 of Supplementary Material) is constituted by the signals at *m*/*z* 325 (M-H-18, H_2_O) and 191 (M-H-152, loss of galloyl). The peak at t_R_ = 29.3 min gave a [M − H]^−^ at *m*/*z* 561; this was assigned to a dimer of the class of condensed tannins composed of catechin and fisetinidol (**9**); fragment ions at *m*/*z* 451, 409, 289 and 271 were observed in its MS/MS spectrum (Figure S4 of Supplementary Material). Quebracho proanthocyanidins consist of a homologous series of oligomers based on the flavan-3-ol structure, with catechin as a starter unit angularly bonded to fisetinidol extender units [28]. In these compounds, the above cited fragment ions are normally present and may be justified by three different mechanisms [29]. In the first mechanism, namely Heterocyclic Ring Fission (HRF), the opening of the heterocyclic ring C occurs with a loss of the A-ring and the release of a fragment ion at *m*/*z* 451 (M-H-110). The second mechanism, Quinone Methide (QM), occurs through the fission of the interflavanyl bond and leads to the formation of a methide quinone; this fragmentation mechanism produces diagnostic ions at *m*/*z* 271 and 289, respectively, due to the two monomers fisetinidol and catechin. The last fragment ion at *m*/*z* 409 (M-H-152) and the derived one at *m*/*z* 391 (M-H-152-18), generated by a consequent loss of water, are originated from the fission of the bonds of the B-ring from the base unit, with the release of a 152 Da unit through a retro-Diels–Alder (rDA). Although the analysis of the fragment ions allows the establishing of the building units and the type of interflavan linkage between these units, it is not possible to discriminate between C-4–C-8 and C-4–C-6 connections, thus, in the case of the catechin-fisetinidol dimer, both structures are reported (**9** and **9′**). 

A poor chromatographic separation on a silica-bonded stationary phase is a common feature of proanthocyanidins, due to the similarity in their structures and to the large number of phenolic groups giving similar interactions with the stationary phase [30,31]. Thus, it is not surprising that largely overlapped peaks are observed between 28 and 36 min. The corresponding polyphenols generated [M − H]^−^ signals at *m*/*z* 643 and 915, which were tentatively assigned to two sulfited oligomers, namely a dimer (**8**) and a trimer (**10**). The presence of sulfited oligomers in commercial tannins is due to industrial treatment, with sodium hydrogen sulfite applied to reduce viscosity and increase tannins solubility in water; the sulfites are formed via cleavage of the pyran ring and the introduction of a C-2 sulfonic acid moiety [31]. The fragmentation patterns (Figure S5 of Supplementary Material) of **8** and **10** encompass signals at M-H-82 (*m*/*z* 561 and 833, respectively), corresponding to the loss of HSO_3_^-^ occurring at C-2 position; signals at m/z 409 and 681 originated from rDA fragmentation of the *m*/*z* 561 and 833, respectively; signals at M-H-354 (*m*/*z* 289 and 561, respectively), corresponding to the loss of a unit of monosulfited fisetinidol. As proof of the obtained data, it has been ascertained that the company that supplied the commercial sample of *Schinopsis lorentzii* tannins has applied these treatments. 

The MS data interpretation of fraction A-2 highlighted the presence of **1**, whose identification was also supported by the HPLC-UV profile (Figure S1 of Supplementary Material). The chromatogram shows between 28 and 44 min a complex multitude of peaks, among which it was possible to identify, in addition to **8** and **10**, a sulfited tetramer at *m*/*z* = 1187 (**20**), with a fragmentation pattern similar to that of the above cited sulfited oligomers. The ^1^H-NMR spectrum of fraction A-2 (Figure 4) shows the presence of three intense signals in the aromatic region at 7.12 (t, *J* = 8.2 Hz, H-5), 6.43 (dd, *J* = 8.2 and 2.1 Hz, H-4/H-6), and 6.37 ppm (bt, *J* = 2.1 Hz, H-2), which were assigned to the aromatic protons of resorcinol (**33**). These data are in agreement with those reported in the literature [32]. The presence of this phenolic compound is attributable to the degradation processes caused by sulfitation [33]. The phenol **33** was not detected in the HPLC-MS analysis, since its molecular weight (110 Da) is lower than the analyzed mass range.

Fraction A-3 shows a TIC profile with some intense peaks. In addition to gallic acid (**1**), whose identification was also supported by the HPLC-UV profile (Figure S1, Supplementary Material), the flavanone eriodictyol (**6**) (t_R_ = 27.7 min, [M − H]^−^ at *m*/*z* 287) was tentatively identified on the basis of two fragment ions at *m*/*z* 269 and 163, ascribable to the loss of water and ring B, respectively. The presence of catechin (**12**) was indicated by the [M − H]^−^ at *m*/*z* 289 (eluted at t_R_ = 37.3 min) and the fragment ions a *m*/*z* 271 (M-H-18, water loss), 245 (M-H-44, CH2=CH-OH loss), 179 (M-H-110, loss of dihydroxybenzene structure), 109 (M-H-179), and 167 (M-H-122), the latter originating from C ring-opening, removal of the B ring in the form of quinone methide (122 Da), and formation of a benzofuranic ring through a benzofuran-forming fission mechanism (BFF) [30]. Furthermore, the fragment at *m*/*z* 137 resulted from a retro-Diels-Alder (rDA) cleavage of ring C, confirmed by the presence of *m*/*z* 151 [34]. Between 36 and 45 min, it was possible to identify the presence of the above cited sulfited tannins **8**, **10**, together with a sulfited pentamer **24** at *m*/*z* 1459. A further peak eluting at t_R_ = 46.2 min, with [M − H]^−^ at *m*/*z* 303, showed an MS/MS spectrum with signals at *m*/*z* 285 (M-H-18), 259 (M-H-44, loss of CO_2_), and 175 (M-H-128, loss of phloroglucinol with an HRF mechanism). These data indicated the presence in this peak of the flavanonol taxifolin (**30**) [35]. In the ^1^H-NMR spectrum of fraction A-3 (Figure 4), the very intense singlet at 7.06 ppm confirmed the presence of gallic acid (**1**). The region of aromatic protons is rich in numerous overlapped signals, which hampered the identification; however, an accurate analysis confirmed the identification of taxifolin (**30**) through some key signals attributable to the protons H-2′ (6.90 ppm, d, *J* = 1.9 Hz), H-5′, and H-6′ (6.79 ppm, m), as well as H-8 (6.28 ppm) and H-6 (6.25 ppm, d, *J* = 1.9 Hz) [34]. Further ^1^H-NMR signals were observed in the region between 5.5 and 2.0 ppm and were attributed to diagnostic signals of eriodictyol (**6**), namely those of protons H-2 (5.14 ppm, dd, *J* = 6.7 and 5.0 Hz), H-3a (2.97 ppm, dd, *J* = 13.9 and 6.7 Hz), and H-3b (2.80 ppm, dd, *J* = 13.9 and 5.0 Hz) of ring C. These attributions were confirmed by the analysis of the COSY spectrum (Figure S6 of Supplementary Material). Eriodictyol is also well known for its antioxidant properties [36]; therefore, the presence of **1** and **6** could justify the marked DPPH scavenging activity observed for this fraction. 

The TIC chromatogram of fraction A-4 (Figure 1) shows an intense peak at t_R_ = 21.1 min, which gave a [M − H]^−^ at *m*/*z* 495, tentatively attributed to an isomer of digalloyl quinic acid. This assumption was confirmed by the analysis of the MS/MS spectrum, where the peaks at *m*/*z* 343 (M-H-152) and 325 (M-H-170), respectively, indicate the loss of one galloyl unit and the loss of gallic acid. The peak at T_R_ = 29.3 min was assigned to the catechin-fisetinidol dimer **9**, according to the above-reported fragmentation pattern. A further peak at t_R_ = 40.4 min, with [M − H]^−^ at *m*/*z* 289, was tentatively attributed to epicatechin (**17**) on the basis of its retention time, different from that of its epimer **12** [37]. An accurate analysis of ^1^H-NMR spectrum of fraction A-4 (Figure 4) confirmed and improved some of the identifications based on MS/MS data. Namely, two isomers of digalloyl quinic acid were identified; the signals at 5.53 (m, H-3), 5.14 (dd, *J* = 7.6, 5.0 Hz, H-5), 2.95 (dd, *J* = 14.0, 5.0 Hz, H-6a) and 2.79 ppm (dd, *J* = 14.0, 7.6 Hz, H-6b) were diagnostic for 3,5-digalloyl quinic acid (**5**), whose identification was corroborated by the analysis of its COSY spectrum (Figure S7 of Supplementary Material), also allowing for an unambiguous assignment of the signals. The signals at 5.68 (m, H-3), 5.22 (dd, *J* = 8.4, 2.8 Hz, H-4), 4.43 (m, H-5), and 2.32 (m, H-2) ppm instead were attributed to 3,4-digalloyl quinic acid (**5′**). Further signals at 5.93 (d, *J* = 1.9 Hz, H-8), 5.86 (d, *J* = 1.9 Hz, H-6), 4.67 (d, *J* = 5.6 Hz, H-2), and 3.97 (m, H-3) were consistent with the presence of epicatechin (**17**), whereas the signals at 4.57 (d, *J* = 5.7 Hz, H-2 fisetinidol), 4.45 (m, H-3 catechin), 4.00 (m, H-3 fisetinidol), 3.09 (dd, *J* = 15.2 and 8.7, H-4a catechin), and 2.66 ppm (dd, *J* = 15.2 and 6.5, H-4b catechin) confirmed the presence of a catechin-fisetinidol dimer. 

The TIC profile of fraction A-5 (Figure 3) shows a series of non-polar peaks eluting between 35 and 60 min. The peak at t_R_ = 37.0 min showed the same [M − H]^−^ and MS/MS fragmentation pattern of **9** found in previous fractions at 29.3 min. This peak was tentatively assigned to a catechin-fisetinidol isomer (**9′**). The peak at t_R_ = 39.2 min showing a [M − H]^−^ at *m*/*z* 647 was attributed to a trigalloyl quinic acid isomer (**11′**), as confirmed by the fragment ions at *m*/*z* 495 (M-H- 152, loss of one unit of galloyl) and *m*/*z* 477 (M-H-170, loss of gallic acid). The complex envelope of overlapped peaks between 40 and 50 min showed several *m*/*z* signals attributable to fisetidinol-catechin oligomers, among which, we report here some identifications. A full characterization of proanthocyanidins was beyond the scope of this work, therefore, these identifications were based only on molecular ions because the adopted MS/MS parameters produced no daughter ions. A summary of these tentatively identified oligomers is reported in Table S1 of Supplementary Material. The ion [M − H]^−^ at *m*/*z* 833 was attributed to a trimer consisting of one unit of catechin and two units of fisetinidol (**23**), with MS/MS fragments at *m*/*z* 681 (M-H-152, loss of B ring by retro-Diels–Alder rearrangement) and *m*/*z* 561 (M-H-272, loss of one fisetinidol unit). Moreover, the peaks [M − H]^−^ at *m*/*z* 1105 (**19**) and *m*/*z* 1122 (**21**), were attributed to tetramers, constituted by one catechin and three fisetinidol units (**19**), and by two catechin and two fisetinidol units (**21**) [23]. The [M − H]^−^ at *m*/*z* 1394 was consistent with a pentamer formed by two catechin and three fisetinidol units (**26**); that at *m*/*z* 1667 was assigned to a hexamer with two catechin and four fisetinidol units (**29**). A trimer consisting of two units of fisetinidol and one of gallocatechin linked by an ether interflavan bond (typical of the A-type structure, Figure 3), was assigned to the [M − H]^−^ at *m*/*z* 847 (**25**). This identification was consistent with the presence of fragments at *m*/*z* 737 (M-H-110), 695 (M-H-152, loss of B ring by retro-Diels-Alder), and 575 (M-H-272, loss of one unit of fisetinidol). Furthermore, a gallocatechin-fisetinidol dimer **31** with an A-type linkage was identified by the presence of a [M − H]^−^ at *m*/*z* 575 and its corresponding MS/MS fragments. As expected, the ^1^H-NMR spectrum of fraction A-5 (Figure 4) shows a complex profile. Two prominent groups of signals in the aromatic region (7.3–5.8 ppm) and in the region typical of sp^3^ methine and methylene protons of dihydropyran moiety (4.7–2.8 ppm), may be attributed to A, B, and C rings of flavan-3-ol units of condensed tannins. Although it was not possible to achieve accurate information regarding these constituents, the large area of these groups of signals is consistent with the presence of proanthocyanidin oligomers as main constituents of this fraction.

Fraction A-6 shows a TIC chromatogram with a low intense peak at t_R_ = 21 min, assigned to the above mentioned digalloyl quinic acid isomers (**5** and/or **5′**). An intense broad peak between 37 and 43 min is the main feature of this fraction; this includes a trigalloyl quinic acid isomer (**11′**) and the oligomers **9′**, **19**, and **23**. The most intense peak gave a [M − H]^−^ at *m*/*z* 285, identified with the flavonoid fisetin (**16**) and confirmed by the fragment ions at *m*/*z* 163 (M-H-122, loss of B ring by a benzofuran-forming fission mechanism (BFF), *m*/*z* 241 (M-H-44, loss of CO_2_), and *m*/*z* 267 (M-H-18, loss of a molecule of water). The ^1^H-NMR spectrum of this fraction is rich in sharp, intense, and well-defined signals in the aromatic region. These signals, precisely at 7.98 (d, *J* = 9.4 Hz, H-5), 7.77 (d, *J* = 1.9 Hz, H-5′), 7.67 (dd, *J* = 8.5 and 1.9 Hz, H-6′), 6.90 (bs, H-2 ′and H-8), and 6.89 ppm (d, *J* = 9.4 Hz, H-6), were attributed to the protons of **16**, which appears to be the most abundant compound of the fraction and is reasonably the main antioxidant constituent in this fraction [25,38]. The high intensity signals at the upper field (1.0–1.5 ppm) may be assigned to polyethylene presumably present as impurity due to the industrial production processes or to storage conditions of the stationary phase.

In fraction A-7, isomers of digalloyl quinic acid (**5** and/or **5′**) and a trigalloyl quinic acid isomer (**11′**) were identified. In the complex envelope of peaks observed between 36 and 42 min, the oligomers **9′**, **19, 21** and **23** and a pentamer (**28**) made up of 1 catechin and 4 fisetinidol units with [M − H]^−^ at *m*/*z* 1377 were identified. The ^1^H-NMR spectrum of this fraction, as well as those of fractions A-8 and A-9, show a complex unresolved cluster of signals, especially in the aromatic region between 7.5 and 6.0 ppm, whose analysis could not provide any useful information (spectra not reported). 

The TIC chromatogram of fraction A-8 (Figure 3), with the highest inhibitory potency towards α-GLU, shows an intense peak at t_R_ = 42.0 min, whose MS analysis indicated the presence of a [M − H]^−^ of *m*/*z* 951 with corresponding fragment ions at *m*/*z* 799 (M-H-152, loss of one galloyl unit) and *m*/*z* 647 (M-H-304, loss of two galloyl units); this was assigned as pentagalloyl quinic acid (**22**). Less intense peaks in this fraction were attributed to a trigalloyl quinic acid (**11**, T_R_ = 32.4 min, [M − H]^−^ at *m*/*z* 647) and a tetragalloyl quinic acid isomer (**14**, T_R_ = 38.2 min, [M − H]^−^ at *m*/*z* 799). The analysis of the complex group of peaks between 36 and 49 min allowed for the identification of the above cited oligomers **9′**, **19**, **21**, and **23** (the profisetinidine series), together with another series of condensed tannins constituted of catechin-3-*O*-gallate linked to variable units of profisetinidol (from 1 to 3) and weighting 152 Da more than the series containing catechin. Hence, the [M − H]^−^ at *m*/*z* 713, 985, and 1121 were attributed to dimer (**15**), trimer (**18**), and tetramer (**27**), respectively.

The TIC profile of fraction A-9 (Figure 3) is characterized by one minor peak between 10 and 18 min and an intense envelope of peaks around 37–52 min. The MS analysis of the former peak allowed the identification of gallic acid methyl ester (**4**) (t_R_ = 15.4 min, [M − H]^−^ at *m*/*z* 183). The intense and only partially overlapped region between 37 and 52 min gave MS signals assigned to the above cited trigalloyl quinic acid isomer (**11′**), tetragalloyl quinic acid (**14**), and oligomers **15**, **18**, **19**, and **23**. The MS analysis of the peak at t_R_ = 37.5 min indicated the presence of a [M − H]^−^ at *m*/*z* 787, identified as tetragalloylglucose (**13**), confirmed by fragment ions at *m*/*z* 617 (M-H-170) and *m*/*z* 635 (M-H-152). The peak at t_R_ = 50.0 min showed a [M − H]^−^ at *m*/*z* = 939, whose fragment ions were at m/z 787 (M-H- 152, loss of galloyl) and *m*/*z* 769 (M-H-170, loss of gallic acid); according to these data, it was assigned as pentagalloylglucose isomer (**32**).

Overall, the HPLC-ESI/MS analysis of A-7–A-9, allowed the identification of hydrolysable tannins (**13**, **32**), esters of quinic acid with different units of gallic acid (**5** and/or **5′**, **11′**, **14**, **22**) and oligomeric condensed tannins (**9′**, **10**, **15**, **18**, **19**, **21, 23**, **27**, **28**), suggesting that these compounds may be responsible for the higher hypoglycemic activity of these fractions. Indeed, α-glucosidase and α-amylase [39] inhibitory activity have been observed to increase, with the degree of polymerization being pentamers more active than monomers [40].

## 3. Materials and Methods 

### 3.1. Chemicals

A sample of commercial tannin Tan’Activ QS SOL (lot n. 03041, SL-T) was provided by Silvateam (S. Michele Mondovì, CN, Italy). Methanol (MeOH), 96% ethanol (EtOH), fluorescein, and Folin–Ciocalteu reagent were purchased from Merck (Darmstadt, Germany); 2,2-diphenylpicrylhydrazyl radical (DPPH•) and formic acid (FA) were obtained from Fluka (Thermo Fischer Scientific, San Jose, CA, USA); quercetin, gallic acid, α-glucosidase from *Saccharomyces cerevisiae* (EC 3.2.1.20, Type I, lyophilized powder, ≥10 units/mg protein; α-GLU), porcine pancreas α-amylase (EC 3.2.1.1, Type VI-B, > 5 units/mg solid; α-AMY), *p*-nitrophenyl-α-d-glucopyranoside (*p*-NP-α-Glc), starch from potato, 3,5-dinitrosalicylic acid (DNS), sodium potassium tartrate tetrahydrate, acarbose, KH_2_PO_4_, Na_2_HPO_4_ 12 H_2_O, fast red B dye, 6-hydroxy-2,5,7,8-tetramethylchromane-2-carboxyl acid (Trolox), CD_3_OD, D_2_O and Sephadex LH-20 were purchased from Sigma Aldrich (Milan, Italy). Cerium (IV) sulfate and ammonium molybdate were obtained from Carlo Erba (Milan, Italy). The 2,2′-Azobis(2-methylpropionamidine) dihydrochloride (AAPH) was purchased from Acros Organics (Thermo Fischer Scientific, San Jose, CA, USA). Thin layer chromatography (TLC) was carried out using pre-coated silica gel F254 plates (Merck, Darmstadt, Germany); spots were visualized under UV light at wavelengths of 254 and 366 nm, or by staining with a solution of cerium sulfate and phosphomolybdic acid followed by heating; or with DPPH or fast red B solutions. HPLC-grade water and acetonitrile (ACN) were purchased from Sigma Aldrich (Milan, Italy). 

### 3.2. HPLC/ESI-MS/MS Analysis

Mass spectrometric analysis was performed on an ion trap mass spectrometer equipped with an electrospray ion source ESI (LCQ-DECA, Thermo Fischer Scientific, San Jose, CA, USA). The mass spectrometer was coupled online with a and autosampler (Thermo Fischer Scientific, San Jose, CA, USA) and a LC-pump (Surveyor MS Pump, Thermo Fischer Scientific, San Jose, CA, USA). Samples were dissolved in methanol ((25 µg/µL) and 5µL were loaded onto a Waters Symmetry RP-C18 column (150 mm × 1 mm i.d., 100 Å, 3.5 µm). Separation was achieved thermosetting the column at 25 °C with a linear gradient of H_2_O + 1% FA and ACN + 1% FA at 50 μL/min. Elution was performed, increasing solvent B from 5% to 15% in 25 min, 25% in 40 min, 30% in 45 min, and 55% in 55 min. Full scan mass spectra were acquired in negative ion mode in the m/z range 150–2000. ESI ion source operated with 220 °C capillary temperature, 30 a.u. sheath gas, −3.5 kV source voltage and −18 V capillary voltage. Mass spectrometric analysis was performed by the data-dependent method with normalized collision energy of 30 a.u. and activation Q set as 0.250. Mass calibration was achieved with a standard mixture of caffeine (Mr 194.1 Da), MRFA peptide (Mr 524.6 Da), and Ultramark (Mr 1621 Da). Data acquisition and data analyses were performed with the Xcalibur v. 1.3 Software.

### 3.3. NMR Analysis

^1^H and gCOSY NMR spectra were acquired on a Varian 500 VNMR-S spectrometer (Varian, Milan, Italy) operating at 499.86 MHz (^1^H) at 300 K and performed using software provided by the manufacturers. Samples were dissolved in CD_3_OD or D_2_O. Chemical shifts (δ) were indirectly referred to TMS using the residual solvent signal as a reference. The pre-sat technique was used, at the occurrence, to suppress the undesired signal of residual water.

### 3.4. Preparation of Tannin Extract (CSE)

Commercial tannin SL-T powder (10.7 g) was extracted three times with ethyl acetate (100 mL) at 25 °C under stirring for a total of 6 h. The filtrates were evaporated until dry, providing a residue (SL-A) of 3.7218 g (34.7%). 

### 3.5. Sephadex LH-20 Fractionation 

The SL-A extract (3.0282 g) was fractionated onto Sephadex LH-20 column (60 x 3 cm), eluted first with water (800 mL) and after with 20% (250 mL), 40% (100 mL), 80% (150 mL), and 100% MeOH (2000 mL) in water; next, the eluents were 25% (150 mL), 50% (150 mL), and 100% acetone (150 mL) in MeOH. Column eluates were pooled according to TLC analysis in nine fractions: A-1 (0.1101 g), A-2 (0.0876 g), A-3 (0.1821 g), A-4 (0.1326 g), A-5 (0.5968g), A-6 (0.4303 g), A-7 (0.8807 g), A-8 (0.1582 g), and A-9 (0.2503 g), with a total weight of 2.8287 g (93% of total extract recovered).

### 3.6. Determination of Total Phenols (GAE)

The total phenols in the SL-T, SL-A and A-1–A-9 fractions were determined with the Folin–Ciocalteu method [41]. Extracts and fractions were dissolved in MeOH solutions or mixture MeOH: H_2_O (50:50) (0.5 mg/mL) and mixed with Folin–Ciocalteu’s reagent (250 μL) and 1.9 M Na_2_CO_3_ solution (500 μL) in a 5 mL volumetric flask. Solutions were incubated at 25 °C for 2 h. The absorbance of each solution was measured at 750 nm with a Jasco V630 spectrometer. A gallic acid standard curve was obtained with different gallic acid concentrations prepared in triplicate (40, 60, 80, and 100 mg/L; r^2^ = 0.995). Results, obtained as mean ± SD, were reported as mg of gallic acid per g of extract/fraction (gallic acid equivalent, GAE). 

### 3.7. DPPH Radical Scavenging Activity Assay

The radical scavenging activity was determined with a DPPH• stable radical, as previously reported [42]. The samples were examined at three different concentrations. Briefly, 10, 20, and 30 µL of solutions of extract or fractions (0.2–0.5 mg/mL) were added to 2 mL of a freshly prepared DPPH• solution (10^−4^ M). The mixtures were incubated for 2 h in the dark at 25 °C, and the absorbance was measured at 515 nm with a Jasco V630 spectrometer. The percentage of reacted DPPH• was calculated according to this equation:

$$quenched\;DPPH•\left(\%\right)=\frac{\left(A_{0}-A_{sample}\right)}{A_{o}}\times 100$$

where A_0_ is the absorbance measured for the DPPH solution; A_sample_ is the absorbance measured for DPPH solution treated with tested compounds. SC_50_ (50% scavenging concentration) is the concentration (µg/mL) of the extract quenching 50% of the initial DPPH• radicals. SC_50_ was calculated from the linear regression between the % of DPPH• quenched and the sample concentration.

### 3.8. Determination of Oxygen Radical Absorbance Capacity (ORAC)

The ORAC of the SL-T and SL-A extracts and A-1–A-9 fractions were measured according to a previously described method [11]. Extracts and fractions were dissolved in water or methanol at 0.1 mg/mL and subsequently, diluted with phosphate buffer (50 mM, pH 7.4) to get final working solutions in the range 0.125–0.0125 mg/mL. Trolox, fluorescein, and AAPH were freshly dissolved in phosphate buffer. A set of four solutions of Trolox in phosphate buffer (6.25, 12.5, 25.0, and 33.3 µmol/L) were prepared to construct a calibration curve (r^2^ = 0.997). The mixtures were prepared into a 96-well microplate. In total, 25 µL of sample solutions, Trolox or phosphate buffer (blank) was added in each well followed by 150 µL of 1 × 10^−7^ M fluorescein solution. The mixtures were shaken at 37 °C for 10 min. The fluorescence of each well was measured immediately after the addition of 25 µL of AAPH (0.153 M) and monitored every 1 min for 46 cycles using a microplate reader (Synergy H1 microplate reader, BioTek, Bad Friedrichshall, Germany) set at λ_Ex_ = 485 nm and λ_Em_ = 528 nm. ORAC values were derived from the linear regression between Trolox concentration and the area under the curve (AUC). The results were expressed as µmol of Trolox equivalents per gram of extract or fraction (µmol TE/g). 

### 3.9. Measurements of α-Glucosidase Inhibition 

The α-glucosidase inhibition assay [10] was performed in a 96-well microplate. Stock solutions of extracts and fractions were prepared in methanol ranging from 1.2 to 0.6 mM. The α-glucosidase solution (0.25 U/mL in 50 mM phosphate buffer, pH 6.8; 100 µL) was mixed with different aliquots (2, 4, 6, 8, 10, 15 µL) of stock solutions. In total, 100 µL of the substrate *p*NP-α-G (78 µM) was added and the microplate was incubated at 37 °C for 30 min under shaking. The absorbance of *p*-nitrophenol released was measured at 405 nm with the Synergy H1 microplate reader after stopping the reaction, by adding 1 M Na_2_CO_3_ solution (10 μL). Acarbose and quercetin were analyzed as reference standards. Each compound was tested at five different concentrations. The amount of methanol used in the experiment did not affect the glucosidase inhibitory activity. The inhibition percentage was calculated by the following equation:

$$inhibition\;\%=\frac{\left(A_{control}-A_{sample}\right)}{A_{control}}\times 100$$

where A_control_ is the absorbance measured for the mixture of enzyme/substrate (without tested compounds); A_sample_ is the absorbance measured in the same conditions and in the presence of the tested compounds. The concentration required to inhibit the 50% activity of the enzyme (IC_50_) was calculated by regression analysis.

### 3.10. Measurements of α-Amylase Inhibition 

The measurement of α-amylase inhibitory activity was performed as previously reported [16]. A stock solution of starch (0.5%) was prepared in 20 mM phosphate buffer (20 mM; pH 6.9) containing 6.7 mM NaCl; the mixture was stirred at 90 °C for 20 min before use. Stock solutions of tested compounds were prepared in water or methanol at concentrations ranging from 5 mg/mL to 0.5 mg/mL. In total, 300 µL of enzyme solution (6 U/mL in phosphate buffer) was added to different aliquots (10, 20, 40, 60, and 100 µL) of the extracts and fractions, and incubated at 37 °C for 10 min. The starch solution (300 µL) was added in the test tubes and the mixtures were incubated at 37 °C for 15 min. The reaction was stopped by adding of 600 µL of 96mM DNS solution (with 30% sodium potassium tartrate in 2N NaOH) and the mixtures were heated in a boiling water bath for 10 min. The test tubes were cooled and diluted with 2 mL of water. The absorbance was measured at 540 nm. Acarbose and quercetin were analyzed as positive references. The control, representing 100% enzyme activity, was carried out by replacing the aliquots of the tested compounds with buffer. The inhibition percentage was calculated by the following equation:

$$inhibition\;\%=\frac{\left(A_{control}-A_{sample}\right)}{A_{control}}\times 100$$

IC_50_ was calculated by regression analysis.

### 3.11. Statistical Analysis 

All experiments were performed in triplicate or quadruplicate, and the results were expressed as mean value ± standard deviations. Principal component analysis (PCA) was performed with the Pareto scaling method and analysis of correlations was determined by the bivariate correlations test. All data were obtained by plotting the experimental measurements on Origin 8.0 software or on Excel 2016. All the obtained results were compared using Analysis of Variance (ANOVA) and differences were designated as statistically significant when *p* < 0.05. 

## 4. Conclusions

Overall, in this work, polyphenol-enriched fractions with antioxidant and/or hypoglycemic activity superior to those of the extract SL-A and the commercial sample obtained from *Schinopsis lorentzii* wood (SL-T) were obtained. The HPLC-ESI/MS-MS and ^1^H-NMR analyses allowed the identification of the main polyphenols responsible for the antioxidant and/or hypoglycemic activity. In particular, fractions A-1 and A-3, with the highest antioxidant activity, include gallic acid (**1**), pyrogallol (**3**), and the flavonols eriodictyol (**6**), catechin (**12**), and taxifolin (**30**), well-known as antioxidant polyphenols. Fractions A-7–A-9, showing promising hypoglycemic activity and good antioxidant capacity, contain a number of condensed (**9′**, **15**, **18**, **19**, **23**, **27**) and hydrolysable tannins (**13**, **32**), as well as esters of quinic acid with different units of gallic acid (**5**, **11**, **11′**, **14**, **22**). In this regard, several literature data report studies of α-GLU and/or α-AMY inhibition by hydrolysable and condensed tannins [16,17,18,43,44], well-known for their antioxidant activity. Although these properties have been reported for esters of caffeic acid with quinic acid [45], this is the first time that gallic acid esters with quinic acid are reported as α-glucosidase and α-amylase inhibitors.

## Figures and Tables

**Table 1:** Percentage weight, gallic acid equivalents (GAE), DPPH scavenging activity, Oxygen Radical Absorbance Capacity (ORAC), and α-glucosidase and α-amylase inhibition activity of the extracts and fractions from *Schinopsis lorentzii* tannins.

Code	Weight %	GAE (mg/g) ^1^	DPPH (SC_50_ ± SD) ^2^	ORAC (TE ± SD) ^3^	α-GLU (IC_50_ ± SD) ^4^	α-AMY (IC_50_ ± SD) ^4^
SL-T	-	303.5 ± 8.8	7.4 ± 0.8	1075.4 ± 71.6	48.9 ± 2.2	129.3 ± 13.0
SL-A	34.7 ^5^	316.3 ± 10.4	5.5 ± 0.6	1410.7 ± 60.8	6.3 ± 0.3	86.1 ± 11.3
A-1	3.9 ^6^	867.5 ± 12.9	4.0 ± 0.6	3345.4 ± 36.3	14.5 ± 1.2	79.5 ± 10.4
A-2	3.2 ^6^	357.3 ± 16.4	7.7 ± 0.7	896.5 ± 11.6	47.2 ± 0.1	66.5 ± 8.9
A-3	6.4 ^6^	756.4 ± 15.3	4.9 ± 0.4	1895.1 ± 23.2	24.9 ± 1.6	81.8 ± 9.3
A-4	4.7 ^6^	467.3 ± 7.2	6.1 ± 1.8	1440.2 ± 16.9	31.6 ± 1.9	294.7 ± 15.9
A-5	21.1 ^6^	475.1 ± 8.5	6.0 ± 0.5	719.0 ± 36.7	22.4 ± 0.3	172.8 ± 13.9
A-6	15.2 ^6^	483.1 ± 19.5	5.3 ± 0.6	1224.9 ± 32.9	8.9 ± 1.0	72.5 ± 6.6
A-7	31.1 ^6^	388.2 ± 5.5	6.1 ± 0.3	793.5 ± 13.7	3.6 ± 0.2	93.6 ± 11.3
A-8	5.6 ^6^	316.7 ± 3.7	6.6 ± 0.5	936.2 ± 28.2	2.1 ± 0.8	64.2 ± 8.4
A-9	8.8 ^6^	279.0 ± 9.1	8.1 ± 0.6	653.6 ± 19.8	2.6 ± 0.3	66.6 ± 7.4
Que	-	-	3.9 ± 0.5	4.8 ± 0.3 ^7^	5.5 ± 0.7	47.6 ± 6.1
Aca	-	-	-	-	97.2 ± 4.8	36.8 ± 9.3

^1^ Results are reported in mg/g of extract or fraction as mean ± SD (n = 3). ^2^ Results are reported in μg/mL of a standard DPPH solution as mean ± SD (n = 3). ^3^ Results are reported in μmol TE/g of extract or fraction as mean ± SD (n = 4).^4^ Results are reported in µg/mL. ^5^ Data are expressed as g/100 g of dried SL-T powder. ^6^ Data are reported as g/100 g of total eluate. ^7^ This value is reported as TE (µM).

**Table 2:** Eigenvectors of the included variables in PCA of Figure 2 on PC1 and PC2.

	Coefficients of PC1	Coefficients of PC2
GAE	0.55081	0.10679
DPPH	0.53842	0.19330
ORAC	0.53726	0.19248
α-GLU	−0.32863	0.57787
α-AMY	−0.10031	0.76174

**Table 3:** Identification by HPLC-ESI-MS/MS and ^1^H-NMR of the main constituents of A-1–A-9 fractions from *Schinopsis lorentzii* tannins.

t_R_ (min)	Identification	Calcd mass	[M − H]^−^	MS/MS Fragments m/z (Relative intensity)	^1^H-NMR Signals δ (multiplicity, *J* = Hz, assignment)	Fraction
3.1	Gallic acid (**1**)	170	169	125 (100)	7.06 (s, H-2/H-6)	A-1–A-3
3.2	Monogalloylglucose isomer (**2**)	332	331	271 (100); 241 (30); 169 (10)		A-1
3.5	Pyrogallol (**3**) ^1^	126	125	-	6.72 (t, *J* = 8.1, H-5), 6.52 *J* = 8.1, H-4/H-6)	A-1
20.0	Gallic acid methyl ester (**4**)	184	183			A-9
21.1	3,5-digalloylquinic acid (**5**) ^2^	496	495	343 (100); 325 (50)	5.53 (m, H-3), 5.14 (bdd, *J* = 7.6, 5.0, H-5); 2.95 (dd, *J* = 14.0, 5.0, H- 6a), 2.79 (dd, *J* = 14.0, 7.6, H-6b)	A-4
21.1	3,4-digalloylquinic acid (**5′**) ^2^	496	495	343 (100); 325 (50)	5.68 (m, H-3), 5.22 (dd, *J* = 8.4, 2.8, H-4) 4.43 (m, H-5), 2.32 (m, H-2)	A-4
27.7	Eriodictyol (**6**)	288	287	269 (100). 163 (20)	5.14 (dd, *J* = 6.7, 5.0, H-2), 2.97 (dd, *J* = 13.9, 6.7, H-3a), 2.80 (dd, *J* = 13.9, 5.0, H-3b)	A-3
28.0	Monogalloylquinic acid isomer (**7**)	344	343	325 (100). 191(40)		A-1
28.3	Dimer isomer (C-SF) (**8**) ^3^	644	643	561 (100;) 409(20;) 289(10)		A-1–A-3
29.3	Dimer isomer (C-F) (**9**) ^2,3^	562	561	543 (20); 541 (40); 409 (100); 289 (60); 271 (30)	4.57 (d, 5.7 Hz, H-2 F), 4.45 (m, H-3 C), 4.00 (m, H-3 F), 3.09 (dd, *J* = 15.2, 8.7, H-4a C), 2.66 (dd, *J* = 15.2, 6.5, H-4b C)	A-1; A-4
29.7	Trimer isomer (C-F-SF) (**10**) ^3^	916	915	833 (100); 681 (20); 561 (20); 289 (20)		A-1–A-3
32.4	Trigalloylquinic acid isomer (**11**)	648	647	495 (100); 477 (20)		A-8
37.0	Dimer isomer (C-F) (**9**′) ^2,3^	562	561	543 (20); 541 (40); 409 (100); 289 (60)		A-5–A-8
37.3	Catechin (**12**)	290	289	271(100); 245(10); 179 (30); 167 (90); 151 (5); 137 (5); 109 (5)		A-3
37.5	Tetragalloylglucose isomer (**13**)	788	787	635 (20); 617 (100)		A-9
38.2	Tetragalloylquinic acid isomer (**14**)	800	799	647 (100); 601 (10); 477 (5); 495 (5)		A-8; A-9
39.2	Trigalloylquinic acid isomer (**11′**) ^2^	648	647	495 (100); 477 (20)		A-5–A-7; A-9
39.3	Dimer isomer (CG-F) (**15**) ^3^	714	713	695 (10); 603 (10); 561 (100); 573 (40); 441 (100)		A-8; A-9
40.3	Fisetin (**16**)	286	285	163 (100); 241 (20); 267 (30)	7.98 (d, *J* = 9.4, H-5), 7.77 (d, *J* = 1.9, H-5′), 7.67 (dd, *J* = 8.5, 1.9, H-6′), 6.90 (bs, H-2′, H-8), 6.89 (d, *J* = 9.4, H-6)	A-6
40.4	Epi-catechin (**17**)	290	289	271(70); 245(100); 179 (30); 167 (60); 151 (5); 137 (5); 109 (5)	5.93 (d, *J* = 1.9, H-8), 5.86 (d, *J* = 1.9, H-6), 4.67 (d, *J* = 5.6, H-2), 3.97 (m, H- 3)	A-4
40.5	Trimer isomer (CG-F-F) (**18**) ^3^	986	985	831 (20); 749 (40); 697 (80); 679 (80); 577 (90); 561 (70); 529 (100)		A-8; A-9
41.1	Tetramer isomer (C-F-F-F) (**19**) ^3^	1106	1105	1086 (20); 995 (40); 953 (100); 935 (60); 833 (70); 561 (20)		A-5–A-9
41.3	Tetramer isomer (C-F-F-SF) (**20**) ^3^	1188	1187	1185 (100); 953 (30); 833 (10)		A-2
41.4	Tetramer isomer (C-C-F-F) (**21**) ^1,3^	1123	1122	-		A-5; A-7; A-8
42.0	Pentagalloylquinic acid isomer (**22**)	952	951	799 (100); 647 (10)		A-8
42.1	Trimer isomer (C-F-F) (**23**) ^3^	834	833	723 (10); 681 (95); 663 (50); 561 (100); 529 (75);289 (10)		A-5–A-9
42.5	Pentamer isomer (C-F-F-F-SF) (**24**) ^2^	1460	1459	1377 (100); 1225 (30); 1105 (30)		A-3
43.3	A-type trimer isomer (GC-F-F) (**25**) ^2^	848	847	737 (40); 695 (100); 575 (60)		A-5
43.3	Pentamer isomer (C-C-F-F-F) (**26**) ^1,3^	1395	1394	-		A-5
43.7	Tetramer isomer (GC-F-F-F) (**27**) ^1,3^	1122	1121	-		A-8
45.0	Pentamer isomer (C-F-F-F-F) (**28**) ^3^	1378	1377	1225 (100); 1207(50);1105 (50); 995 (30); 833 (20)		A-7
45.1	Hexamer isomer (C-C-F-F-F-F) (**29**) ^1,3^	1668	1667	-		A-5
46.2	Taxifolin (**30**)	304	303	285 (100); 259 (40);175 (70)	6.90 (d, *J* = 1.9, H-2′), 6.79 (m, H-5′, H-6′), 6.28 (d, *J* = 1.9, H-8), 6.25 (d, *J* = 1.9, H-6)	A-3
48.1	A-type bond dimer isomer (GC-F) (**31**) ^3^	576	575	533 (20); 467 (30); 437 (25); 425 (40); 409 (50); 289 (100)		A-5
50.0	Pentagalloylglucose isomer (**32**)	940	939	787 (100); 769 (10)		A-9
-	Resorcinol (**33**) ^4^				7.12 (t, *J* = 8.2, H-5), 6.43 (dd, *J* = 8.2, 2.1, H-4/H-6), 6.37 (bt, *J* = 2.1, H-2)	A-2

^1^ These MS identifications were based only on molecular ions because the adopted MS/MS parameters produced no daughter ions. ^2^ These couples of indistinguishable isomers with different retention times were numbered with or without superscript (N, N′). ^3^ Catechin (C); Fisetinidol (F); Gallocatechin (GC); Catechin-3-*O*-gallate (CG); Sulfited Fisetinidol (SF). ^4^ Identification was achieved only by ^1^HNMR data analysis.

## Supplementary Materials

The most significant MS/MS fragmentation patterns of identified compounds, a copy of COSY spectra of A-3 and A-4 fractions and a list of the identified condensed tannin oligomers are available online.
