# Supplementary material for: Mass Spectrometry and 1H-NMR Study of Schinopsis lorentzii (Quebracho) Tannins as a Source of Hypoglycemic and Antioxidant Principles
Source: Molecules. 2020 Jul 17;25(14):3257. doi: 10.3390/molecules25143257 (PMC7397293; doi:10.3390/molecules25143257)
Supplement: Supplementary file 1 [file molecules-25-03257-s001.pdf]

Supplementary Materials for

Mass Spectrometry and ^{1}H-NMR Study of *Schinopsis lorentzii* (Quebracho) Tannins as a Source of Hypoglycemic and Antioxidant Principles

Nunzio Cardullo, Vera Muccilli,* Vincenzo Cunsolo and Corrado Tringali

Department of Chemical Sciences, University of Catania, Viale A. Doria 6, 95125-Catania, Italy; ncardullo@unict.it (N.C.); vcunsolo@unict.it (V.C.); ctringali@unict.it (C.T.)

* Correspondence: v.muccilli@unict.it; Tel.: +39-095-7385041

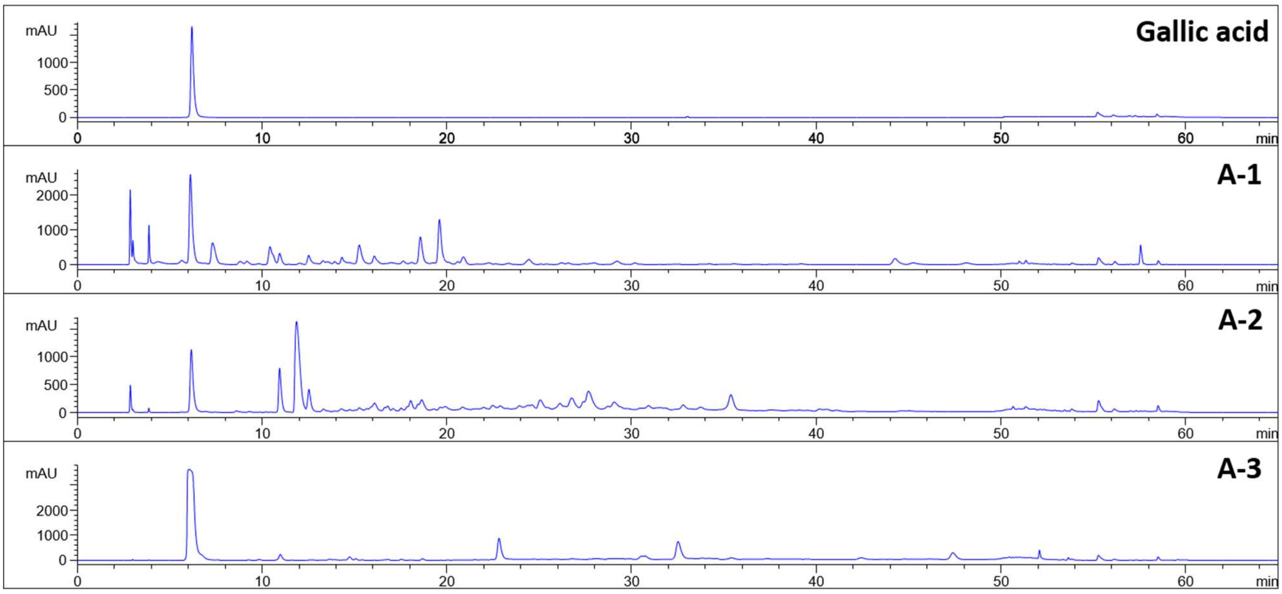

Figure S1. HPLC-UV profiles (280 nm) of gallic acid and fractions A1 - A-4.

HPLC-UV analyses of gallic acid and fractions A1 - A-3

The HPLC-UV chromatograms were carried out using an Agilent Series G1354A pump and an Agilent UV G1315D as diode array detector. An Agilent Series 1100 G1313A autosampler was used for sample injection. The analyses were performed on an analytical reversed phase column (Luna C18, 5 µm; 4.6 × 250 mm; Phenomenex) eluted with a gradient of H2O/H+ (99/1; A)–CH3CN/H+ (99/1; B) at 1 mL/min as follow: t0 min B = 5%, t25 min B = 15%, t40 min B = 25%, t45 min B = 30%, t55 min B = 55%, t60 min B = 90%, t65 min B = 100%, t70 min B = 5%. Gallic acid standard curve was obtained with different gallic acid concentrations prepared in triplicate (40, 60, 80 and 100 mg/L; r2 = 0.991).

Typical MS/MS fragmentation pattern of the different types of tannins identified

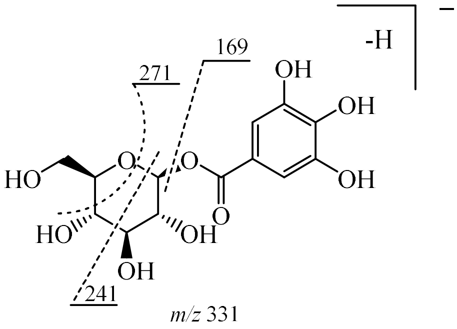

Figure S2. MS/MS fragmentation pattern of [M-H]⁻ at m/z 331 identified as monogalloylglucose isomer (**2**).

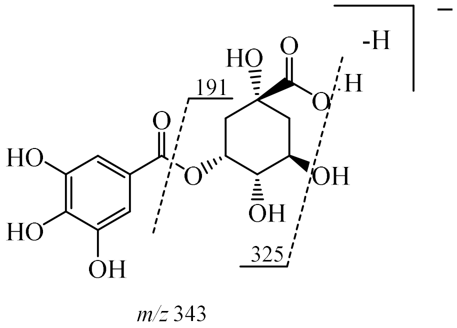

Figure S3. MS/MS fragmentation pattern of [M-H]⁻ at m/z 343 identified as monogalloylquinic acid isomer (**7**).

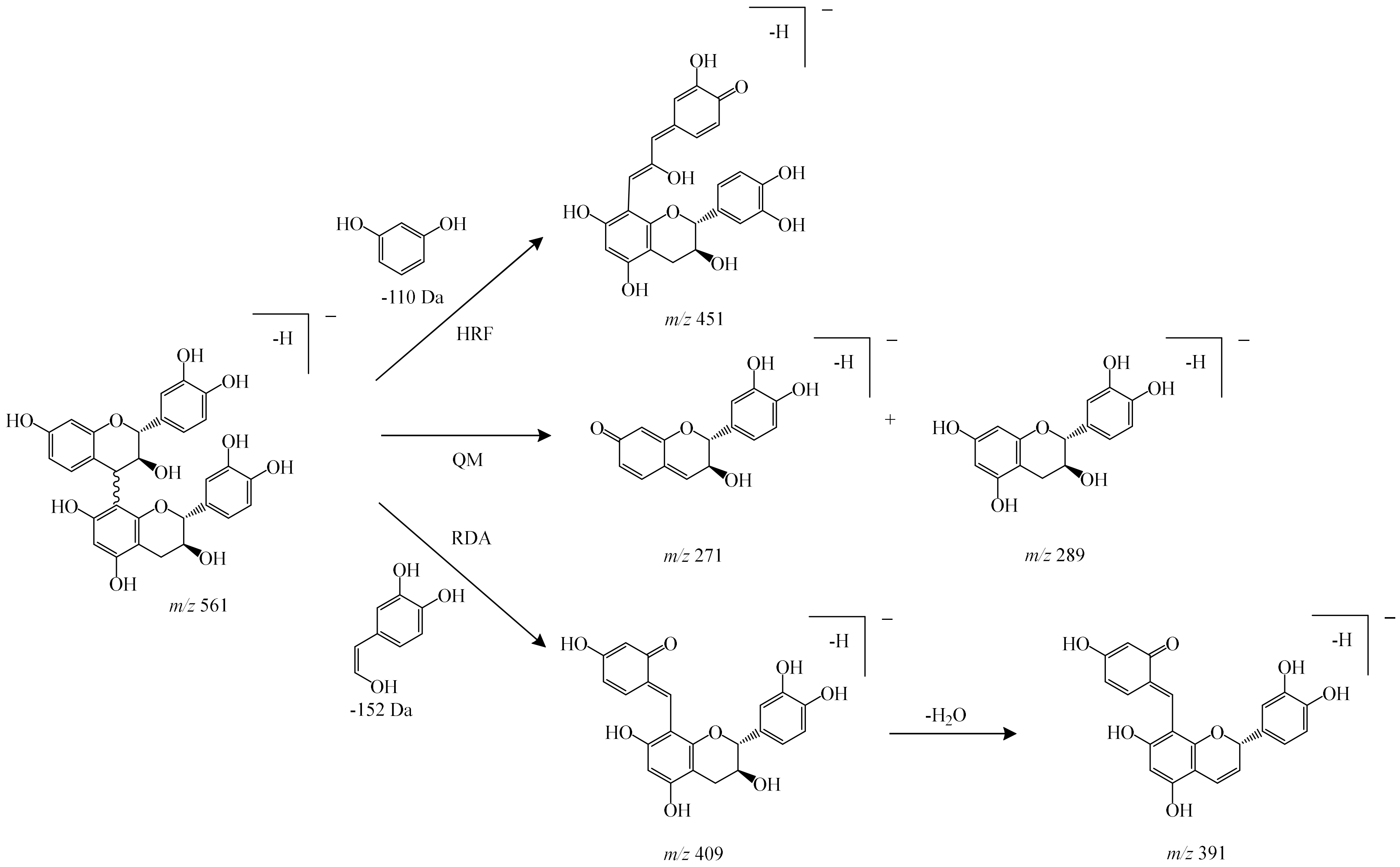

Figure S4. MS/MS fragmentation pattern of $[M-H]^-$ at m/z 561 identified as dimer **9/9'**: HRF (Heterocyclic Ring Fission); QM (quinone methide); rDA (Retro Diels Alder).

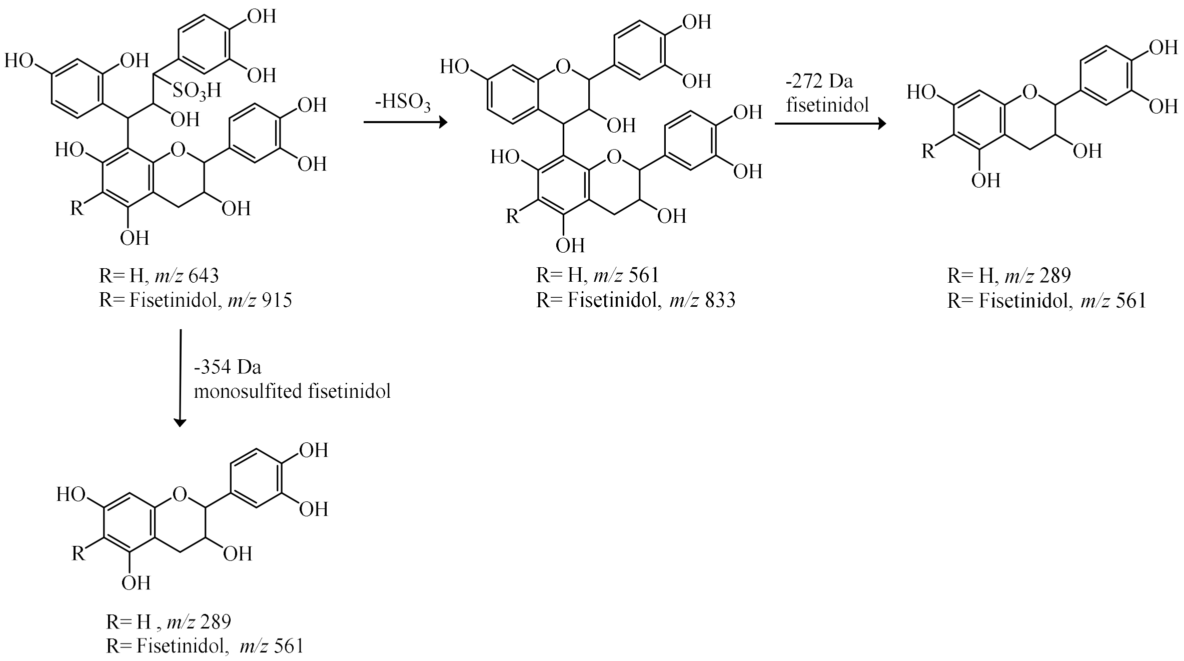

Figure S5. MS/MS fragmentation pattern of [M-H]$^-$ at m/z 643 identified as (**8**) and of [M-H]$^-$ at m/z 915 identified as **10.**

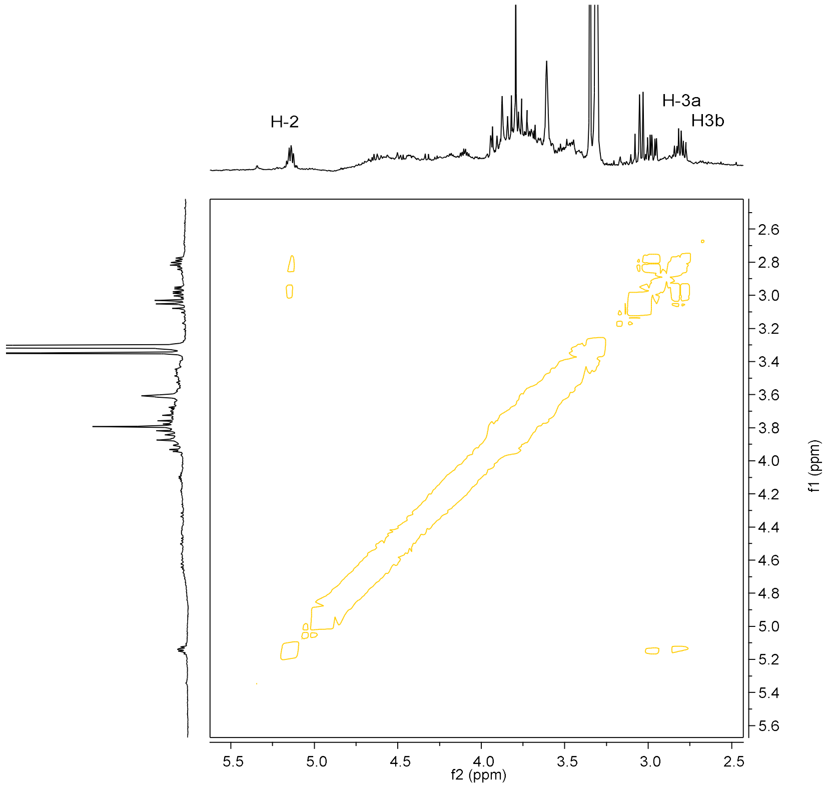

Figure S6. gCOSY spectrum (from 5.5 to 2.5 ppm) of fraction A-3.

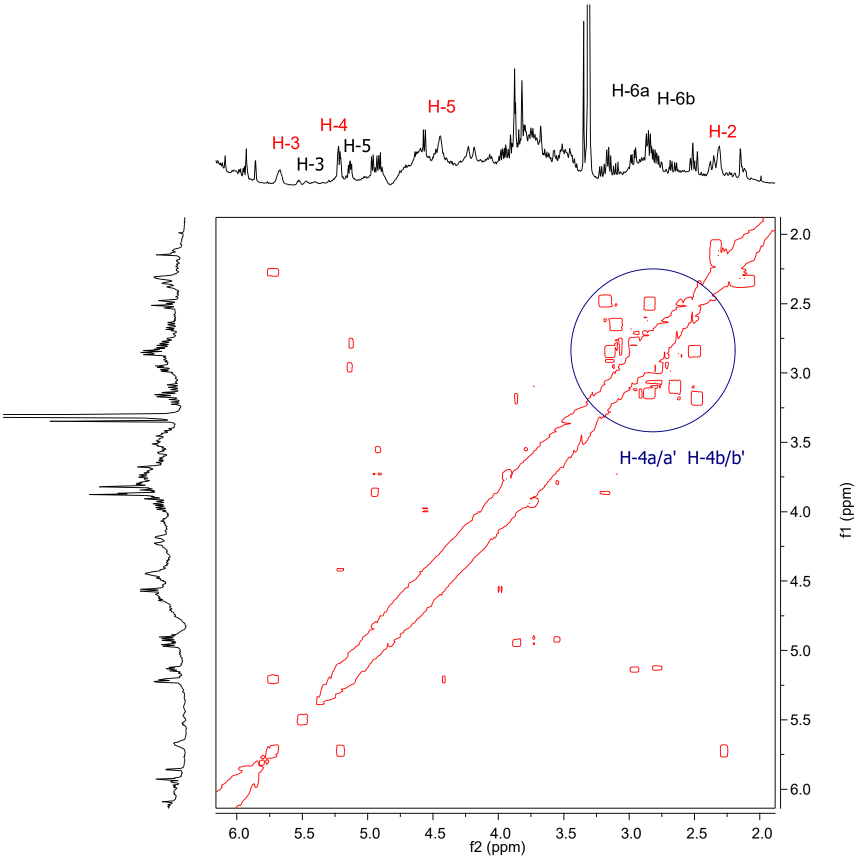

Figure S7. gCOSY spectrum (from 6.0 to 2.0 ppm) of A-4. In dark are reported proton signals of **5** (3,5-digalloyl quinic acid), in red those of **5'** (3,4-digalloyl quinic acid), in blue key COSY correlations of **9/9'**.

Table S1. Structural features of condensed tannin oligomers tentatively identified.

	**Catechin C**	**Gallocatechin GC**	**Catechin-3-*O*-gallate CG**	**Fisetinidol F**	**Sulfited Fisetinidol SF**	**Structure Type**	**MW**	**M-H⁻**
**MW**	290	306	442	273	354			
**Dimer**	1			1		B	562	561
		1		1		A	576	575
			1	1		B	714	713
	1				1	B	644	643
**Trimer**	1			2		B	834	833
		1		2		A	848	847
			1	2		B	986	985
**Tetramer**	1			3		B	1106	1105
		1		3		B	1122	1121
	1			2	1	B	1188	1187
**Pentamer**	1			4		B	1378	1377
	2			3		B	1395	1394
	1			3	1	B	1460	1459
**Hexamer**	2			4		B	1668	1667
